# *Herpesviridae* reactivation for poor outcome in ARDS patients with ECMO: criminal or witness?

**DOI:** 10.1186/s13613-020-0626-4

**Published:** 2020-01-28

**Authors:** Huixue Huang, Hangyong He

**Affiliations:** 1 Department of Internal Medicine, Beijing University of Technology Hospital, Beijing, China; 2 Department of Respiratory and Critical Care Medicine, Beijing Institute of Respiratory Medicine, Beijing Chao-Yang Hospital, Capital Medical University, No. 8 Gongren Tiyuchang Nanlu, Chaoyang District, Beijing, 100020 China

Dear editor,

We read with interest of the report by Hraiech and colleagues [1] about the *Herpesviridae* reactivation among non-immunocompromised critically ill patients with acute respiratory distress syndrome (ARDS) supported with extracorporeal membrane oxygenation (ECMO). They found that *Herpesviridae* reactivation is frequent among patients with severe ARDS under ECMO and is associated with a longer duration of mechanical ventilation. From our point of view, a few other concerns related to who, how and why for the *Herpesviridae* reactivation affects outcome in ARDS could be raised.

First, who has a *Herpesviridae* reactivation should be focused? In Hraiech’s study [1], they determined the frequency of *Herpesviridae* reactivation and its impact on prognosis only in patients during ECMO for severe ARDS. However, *Herpesviridae* reactivation occurring in all ARDS patients should be investigated. Within all ARDS patients, by comparing the reactivation related to ARDS with no-ECMO support, and the reactivation after ECMO insertion, whether a *Herpesviridae* reactivation was worsening under ECMO could be confirmed.

The second question is how a *Herpesviridae* reactivation or active infection should be defined in ARDS patients? In Hraiech’s report [1], Herpes Simplex Virus (HSV) reactivation was diagnosed mainly by throat or BAL samples, and only 3 patients (5%) exhibited a positive HSV viremia, and cytomegalovirus (CMV) reactivation was diagnosed mainly from blood PCR. However, whether a positive PCR from the airway sample should be treated as a reactivation or as an evidence of infection is still debatable [2]. Previous study [3, 4] found that seropositivity for Epstein–Barr virus (EBV), one of *Herpesviridae*, was associated with mortality of ICU patients with respiratory failure. Thus, when looking for the association of reactivation and the outcome, we suggested that only patients with a seropositivity for *Herpesviridae* should be included. Furthermore, Hraiech and colleagues [1] found that HSV reactivation occurred earlier than CMV reactivation. In our study, airway sample may show an earlier and higher positivity than serum samples for EBV [3]. Therefore, the time of reactivation for HSV and CMV should be compared for samples from serum and airway, respectively.

Finally, why *Herpesviridae* reactivation caused a poor outcome in ARDS patients with ECMO support is still unclear. On the one hand, *Herpesviridae* reactivation may indicate an active infection and related damage to the organ function, which lead to a poor prognosis. However, in Hraiech’s study [1], the definition of reactivation by a positive airway sample could not interpreted as true infection. And they reported that alveolar procollagen III level, an indicator of pulmonary fibrosis, was not different between both groups. These results could not support that reactivation leads a poor outcome by infection. On the other hand, in our previous report [3], the seropositivity for EBV associated with mortality maybe correlated with a low CD3+ CD8+ T cell count. Thus, the biomarkers of an ARDS or sepsis-induced immunoparalysis should be explored as a possible mechanism of reactivation and poor outcome [5].

Overall, whether *Herpesviridae* reactivation is directly responsible for worse clinical outcomes or if it is a consequence and a witness of the severity of the disease, as in ARDS populations with/without ECMO, still needs further investigation.

## Data Availability

Not applicable.

## Abbreviations

ARDS: acute respiratory distress syndrome
CMV: cytomegalovirus
EBV: Epstein–Barr virus
ECMO: extracorporeal membrane oxygenation
HSV: Herpes Simplex Virus
